# Food matters: Anorexia nervosa and the microbiome: First findings of a European cooperation

**DOI:** 10.1192/j.eurpsy.2021.100

**Published:** 2021-08-13

**Authors:** B. Herpertz-Dahlmann, J. Seitz, R. Adan, S. Fetissov, J. Baines, A. Karwautz, A. Van Elburg

**Affiliations:** 1 Department Of Child And Adolescent Psychiatry, RWTH Aachen University, Aachen, Germany; 2 Translational Neuroscience, UMCU, Utrecht, Netherlands; 3 Faculte Des Sciences, Inserm UMR 1239, Rouen, France; 4 Institute For Experimental Medicine, Christian´s Albrecht University of Kiel, Kiel, Germany; 5 Department Of Child And Adolescent Psychiatry, Medical University Vienna, Wien, Austria; 6 Adolescent Eating Disorders, Altrecht Eating Disorders Rintveld, Zeist and Department of Social Sciences, University of Utrecht, Utrecht, Netherlands

**Keywords:** low grade inflammation, anorexia nervosa, Microbiome, body weight

## Abstract

Anorexia nervosa (AN) is one of the most common chronic disorders in adolescence with still high mortality rates. Knowledge on gut-brain interaction might help to develop new treatments, as severe starvation-induced changes of the microbiome in AN-patients have been demonstrated, which do not alleviate with weight gain. In our own pilot study alpha-diversity was increased in patients with AN after short-term weight recovery, while beta diversity showed clear group differences with healthy controls before and after weight gain. A reduction of taxa belonging to Enterobacteriaceae at admission and discharge and an increase in taxa belonging to Lachnospiraceae at discharge were typically found in patients with AN. The work plan of our European project comprises an observational study and two phase II RCTs with the application of omega-3-PUFA and a multistrain psychobiotic to both, humans and rodents. With the help of a well-established animal model for AN (activity-based anorexia, ABA), the effect of stool transplants from patients to rodents will be analysed. Longitudinal MRI will be conducted in rodents together with cellular and molecular brain analyses. In addition, immune response and circulating antibodies associated with the presence of certain bacterial strains and interaction with hunger and satiety hormones will be explored. We hope that by this translational research we may systematically investigate the role of an altered microbiome for the course of AN and to identify new therapeutic tools.

**Disclosure:**

This project is funded by ERA-NET of the European Union.

